# Supplementary material for: ProteinShader: illustrative rendering of macromolecules
Source: BMC Struct Biol. 2009 Mar 30;9:19. doi: 10.1186/1472-6807-9-19 (PMC2672931; doi:10.1186/1472-6807-9-19)
Supplement: Additional file 1 — ProteinShader program without source code. This compressed file contains the complete ProteinShader program including associated libraries, but no source code. A README.txt file gives an overview of the ProteinShader distribution, and the index.html file in the help subdirectory has directions on getting started with the program as well as a set of tutorials. [file 1472-6807-9-19-S1.zip › ProteinShader-beta-0_9_4-binary/help/api/org/proteinshader/structure/enums/class-use/DecorationEnum.html]

Uses of Class org.proteinshader.structure.enums.DecorationEnum (ProteinShader API)


|  |  |  |
| --- | --- | --- |
| **Overview** \| **Package** \| **Class** \| **Use** \| **Tree** \| **Deprecated** \| **Index** \| **Help** | |  |
| PREV   NEXT | **FRAMES**    **NO FRAMES**     **All Classes** |  |


---


## **Uses of Class org.proteinshader.structure.enums.DecorationEnum**

| Packages that use DecorationEnum | |
| --- | --- |
| **org.proteinshader.gui.components.controlpanel** | Holds the JPanels used in the right-side retractable control panel of the GUI. |
| **org.proteinshader.structure** | Holds the classes that store information from a Protein Data Bank file: Structure, Model, Chain, AminoAcid, Heterogen, Water, Atom, Bond, Helix, BetaStrand, Loop, *etc*. |
| **org.proteinshader.structure.enums** | Holds the enums needed by org.proteinshader.structure and its subpackages. |
| **org.proteinshader.structure.visitor.modifiers** | Holds helper classes for the ModifierVisitor class, which is used to modify Drawable objects (Atoms, Bonds, and Segments). |

Uses of DecorationEnum in org.proteinshader.gui.components.controlpanel

| Methods in org.proteinshader.gui.components.controlpanel with parameters of type DecorationEnum | |
| --- | --- |
| `void` | `DecorationsPanel.setDecoration(DecorationEnum decoration)`             Sets up a SegmentModifier to change the decoration type and then passes the SegmentModifier to the RadioPanel. |

Uses of DecorationEnum in org.proteinshader.structure

| Fields in org.proteinshader.structure declared as DecorationEnum | |
| --- | --- |
| `static DecorationEnum` | `Segment.DECORATION`             The default decoration is HALFTONING. |

| Methods in org.proteinshader.structure that return DecorationEnum | |
| --- | --- |
| `DecorationEnum` | `Segment.getDecoration()`             Returns the decoration type for this Segment (PLAIN, WIREFRAME, TEXT\_LABEL, PATTERNS, or HALFTONING). |

| Methods in org.proteinshader.structure with parameters of type DecorationEnum | |
| --- | --- |
| `void` | `Segment.setDecoration(DecorationEnum decoration)`             Sets the decoration type for this Segment (PLAIN, WIREFRAME, TEXT\_LABEL, or HALFTONING). |

Uses of DecorationEnum in org.proteinshader.structure.enums

| Methods in org.proteinshader.structure.enums that return DecorationEnum | |
| --- | --- |
| `static DecorationEnum` | `DecorationEnum.valueOf(String name)`             Returns the enum constant of this type with the specified name. |
| `static DecorationEnum[]` | `DecorationEnum.values()`             Returns an array containing the constants of this enum type, in the order they're declared. |

Uses of DecorationEnum in org.proteinshader.structure.visitor.modifiers

| Methods in org.proteinshader.structure.visitor.modifiers with parameters of type DecorationEnum | |
| --- | --- |
| `void` | `SegmentModifier.setDecoration(DecorationEnum decoration)`             Stores a memory that the decoration (PLAIN, WIREFRAME, TEXT\_LABEL, or HALFTONING) needs to be set on the Segment. |

---


|  |  |  |
| --- | --- | --- |
| **Overview** \| **Package** \| **Class** \| **Use** \| **Tree** \| **Deprecated** \| **Index** \| **Help** | |  |
| PREV   NEXT | **FRAMES**    **NO FRAMES**     **All Classes** |  |


---

# *Copyright © 2007-2008*
